# *mer*-Bis[3,5-difluoro-2-(2-pyrid­yl)phenyl-κ^2^ *C* ^1^,*N*]{5-(2-pyridyl-κ*N*)-3-[3-(4-vinyl­benz­yloxy)phen­yl]-1,2,4-triazol-1-ido}iridium(III) methanol solvate

**DOI:** 10.1107/S1600536809052726

**Published:** 2009-12-16

**Authors:** Peter G. Jones, Marc Debeaux, Andreas Weinkauf, Henning Hopf, Wolfgang Kowalsky, Hans-Hermann Johannes

**Affiliations:** a Institut für Anorganische und Analytische Chemie, Technical University of Braunschweig, Postfach 3329, 38023 Braunschweig, Germany; b Labor für Elektrooptik am Institut für Hochfrequenztechnik, Technical University of Braunschweig, Postfach 3329, 38023 Braunschweig, Germany; c Institut für Organische Chemie, Technical University of Braunschweig, Postfach 3329, 38023 Braunschweig, Germany

## Abstract

In the title compound, [Ir(C_11_H_6_F_2_N)_2_(C_22_H_17_N_4_O)]·CH_3_OH, the coordination at iridium is essentially octa­hedral, but with distortions associated with the bite angles of the ligands [76.25 (9)–80.71 (12)°] and the differing *trans* influences of C and N ligands [Ir—N = 2.04 Å (average) *trans* to N but 2.14 Å *trans* to C]. All three bidentate ligands have coordinating ring systems that are almost coplanar [inter­planar angles = 1.7 (1)–3.8 (2)°]. The vinyl­benzyl group is disordered over two positions with occupations of 0.653 (4) and 0.347 (4). The methanol solvent mol­ecule is involved in a classical O—H⋯N hydrogen bond to a triazole N atom.

## Related literature

For background to organic light-emitting diodes (OLEDs), see: Adachi *et al.* (2001 ▶); Baldo *et al.* (1998 ▶); Burroughes *et al.* (1990 ▶); Chang *et al.* (2007 ▶); Coppo *et al.* (2004 ▶); Dedeian *et al.* (1991 ▶); Dixon *et al.* (2000 ▶); Gong *et al.* (2002 ▶); Grushin *et al.* (2001 ▶); Lamansky *et al.* (2001 ▶); Schütz *et al.* (2008 ▶); Suzuki *et al.* (2005 ▶); Tang & VanSlyke (1987 ▶); You & Park (2005 ▶). 
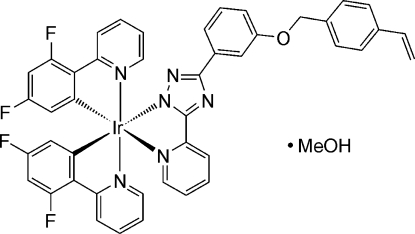


## Experimental

### 

#### Crystal data

[Ir(C_11_H_6_F_2_N)_2_(C_22_H_17_N_4_O)]·CH_4_O
*M* *_r_* = 957.97
Triclinic, 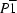

*a* = 9.8934 (1) Å
*b* = 12.3039 (2) Å
*c* = 16.8933 (3) Å
α = 81.7429 (14)°
β = 83.2858 (11)°
γ = 69.9647 (14)°
*V* = 1906.82 (5) Å^3^
*Z* = 2
Mo *K*α radiation
μ = 3.57 mm^−1^
*T* = 100 K
0.20 × 0.15 × 0.10 mm

#### Data collection

Oxford Diffraction Xcalibur E diffractometer
Absorption correction: multi-scan (*CrysAlis RED*; Oxford Diffraction, 2008 ▶) *T*_min_ = 0.848, *T*_max_ = 1.000
53102 measured reflections
10448 independent reflections
8442 reflections with *I* > 2σ(*I*)
*R*_int_ = 0.034

#### Refinement

*R*[*F* ^2^ > 2σ(*F* ^2^)] = 0.029
*wR*(*F* ^2^) = 0.064
*S* = 0.93
10448 reflections
538 parameters
69 restraints
H-atom parameters constrained
Δρ_max_ = 2.18 e Å^−3^
Δρ_min_ = −0.84 e Å^−3^

### 

Data collection: *CrysAlis CCD* (Oxford Diffraction, 2008 ▶); cell refinement: *CrysAlis RED* (Oxford Diffraction, 2008 ▶); data reduction: *CrysAlis RED*; program(s) used to solve structure: *SHELXS97* (Sheldrick, 2008 ▶); program(s) used to refine structure: *SHELXL97* (Sheldrick, 2008 ▶); molecular graphics: *XP* (Siemens, 1994 ▶); software used to prepare material for publication: *SHELXL97*.

## Supplementary Material

Crystal structure: contains datablocks I, global. DOI: 10.1107/S1600536809052726/tk2595sup1.cif

Structure factors: contains datablocks I. DOI: 10.1107/S1600536809052726/tk2595Isup2.hkl

Additional supplementary materials:  crystallographic information; 3D view; checkCIF report

## Figures and Tables

**Table 1:** Selected bond lengths (Å)

Ir—C11	2.010 (3)
Ir—C22	2.012 (3)
Ir—N1	2.033 (2)
Ir—N2	2.049 (2)
Ir—N4	2.118 (2)
Ir—N3	2.158 (2)

**Table 2:** Hydrogen-bond geometry (Å, °)

*D*—H⋯*A*	*D*—H	H⋯*A*	*D*⋯*A*	*D*—H⋯*A*
O99—H99⋯N6	0.84	2.04	2.853 (3)	164
C24—H24⋯O99	0.95	2.49	3.368 (4)	154
C31—H31⋯O99	0.95	2.66	3.587 (4)	164

**Fig. 1.**
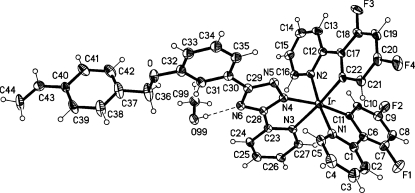
Structure of the title complex. Displacement ellipsoids represent 50% probability levels. The dashed line represents the classical H bond H99···N6. The minor disorder component is omitted.

**Fig. 2.**
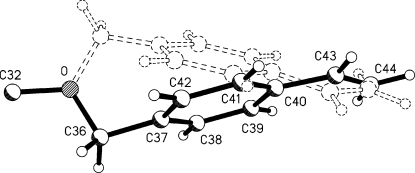
Detail of the structure, showing both disorder components of the vinylbenzyl group. Atoms C32 and O are ordered. The minor component is shown as dashed bonds/atoms. Only the major component is labelled.

## supplementary crystallographic information

### Comment

Since Tang and VanSlyke (1987) reported on the first organic light-emitting diode (OLED) based on the electroluminescence of tris(8-hydroxyquinoline)aluminium, and Burroughes *et al.* (1990) discovered a device with an electroluminescent organic polymer (PLED), OLEDs have attracted much attention because of their application in flat-panel and large-area displays (Burroughes *et al.*, 1990; Tang & VanSlyke, 1987). By incorporation of phosphorescent organometallic complexes of transition metals with a strong spin-orbit coupling, electroluminescent quantum efficiencies up to 100% can be achieved (Baldo *et al.*, 1998; Gong *et al.*, 2002). Among these heavy metals, iridium(III)-based complexes are especially attractive because of their highly tunable emission colors and relatively short phosphorescence lifetimes (Dixon *et al.*, 2000; Lamansky *et al.*, 2001).

Homo- and heteroleptic iridium(III) complexes are versatile and readily available. The emission colour of heteroleptic complexes can be tuned by varying the two monoanionic cyclometalating ligands (Dedeian *et al.*, 1991; Grushin *et al.*, 2001) and/or the third ligand (Chang *et al.*, 2007; You *et al.*, 2005). For example, the replacement of the picolinate by a triazolylpyridine ligand in the well known "blue" emitting FIrpic leads to a hypsochromically shifted phosphorescence (Adachi *et al.*, 2001; Coppo *et al.*, 2004).

In terms of large scale fabrication, the easiness and inexpensiveness of wet processes such as spin coating, dip coating or ink-jet printing of PLEDs represent important advantages over vacuum techniques. Chemically attaching emitter moieties to the polymer matrix can prevent degradation processes clearly caused by phase separation (Suzuki *et al.*, 2005). Furthermore, the covalent attachment also prevents cascading energy transfer through steric shielding (Schütz *et al.*, 2008).

In this contribution, we report the structure of a styrene-functionalized, cyan-emitting complex that can be incorporated in an ambipolar polymer approach (Suzuki *et al.*, 2005). The structure of the title complex, which crystallizes as a methanol solvate, is shown in Fig. 1. The vinylbenzyl group is disordered over two positions with occupations 0.653 (4), 0.347 (4); this is shown in Fig. 2. The coordination at iridium is essentially octahedral, but with some distortions associated with the restricted bite of the bidentate ligands; C11—Ir—N1 80.20 (11), C22—Ir —N2 80.71 (12), and N4—Ir—N3 76.25 (9)°. All five-membered chelate rings are planar (max. r.m.s.d. 0.019 Å) and mutually perpendicular (max. deviation 3°). Interplanar angles between the coordinating rings of each ligand are small: 3.8 (2)° in the ligand based on N1/C11, 3.4 (2)° for N2/C22, and 1.7 (1)° for N3/N4.

The bond lengths, Table 1, at Ir reflect the different *trans* influences of C and N ligands, although the significant difference between the two Ir—N bond lengths *trans* to C has no obvious explanation.

Hydrogen bonds are listed in Table 2. The methanol molecule is linked to an N atom of the triazole ring *via* a classical H bond. The contacts H24···O99 and H31···O99 may also be interpreted as weak H bonds within the asymmetric unit, but are not drawn explicitly in Fig. 1.

### Experimental

A suspension of bis[3,5-difluoro-2-(pyridin-2-yl-κ*N*)phenyl]-[3-(3-hydroxyphenyl)-5-(pyridin-2-yl-κ*N*)-1,2,4-triazol-1-yl]-iridium(III) (400 mg, 494 µmol) in dry DMF (5 ml) was treated with sodium hydride (60% dispersion in mineral oil, 40 mg, 988 µmol) and stirred for 30 min at room temperature. 1-(Chloromethyl)-4-vinylbenzene (151 mg, 988 µmol) was added and stirred for 1 d at room temperature. The solvent was removed under reduced pressure at 373 K. The product was isolated from the residue by flash chromatography on silica gel (dichloromethane/acetone 4:1; *R*_f_ = 0.40) as a yellow solid (255 mg, 56%). Single crystals were grown from dichloromethane/methanol solution. Elemental analysis: calculated for C_44_H_29_F_4_IrN_6_O: C 57.07, H 3.16, N 9.08%; found: C 57.06, H 3.04, N 8.82%.

### Refinement

Methyl- and hydroxyl-H atoms were identified in a difference synthesis and refined as idealized rigid groups allowed to rotate but not tip (C—H 0.98, O—H 0.84 Å, H—C—H and C—O—H angles 109.5°). These *U*(H) values were fixed at 1.5 × *U*_eq_(C) of the parent C or O atom. Other hydrogen atoms were included at calculated positions using a riding model with C—H distances in Å as follows; aromatic C—H and C═CH_2_ 0.95, methylene 0.99. These *U*(H) values were fixed at 1.2 × *U*_eq_(C) of the parent C atom.

The atoms C36–C44 (the vinylbenzyl group) are disordered over two positions rotated about the O—C32 bond, with refined occupancies 0.653 (4) and 0.347 (4). The less occupied group was refined isotropically. For the disordered group, rigid idealized aromatic rings (C—C 1.395, C—H 0.95 Å and all angles 120°), similarity restraints and and a system of restraints to *U* values were employed to improve refinement stability, but nevertheless the dimensions of the disordered group should be interpreted with caution.

There are several peaks of 1.5–2.2 e Å^-3^*ca* 0.8 Å from the Ir atom; these may reasonably be attributed to residual absorption errors.

### Crystal data

**Table:**

[Ir(C_11_H_6_F_2_N)_2_(C_22_H_17_N_4_O)]·CH_4_O	*Z* = 2
*M**_r_* = 957.97	*F*(000) = 948
Triclinic, *P*1	*D*_x_ = 1.668 Mg m^−^^3^
Hall symbol: -P 1	Melting point: 560 K
*a* = 9.8934 (1) Å	Mo *K*α radiation, λ = 0.71073 Å
*b* = 12.3039 (2) Å	Cell parameters from 25891 reflections
*c* = 16.8933 (3) Å	θ = 2.0–30.7°
α = 81.7429 (14)°	µ = 3.57 mm^−^^1^
β = 83.2858 (11)°	*T* = 100 K
γ = 69.9647 (14)°	Prism, yellow
*V* = 1906.82 (5) Å^3^	0.20 × 0.15 × 0.10 mm

### Data collection

**Table:**

Oxford Diffraction Xcalibur E diffractometer	10448 independent reflections
Radiation source: Enhance (Mo) X-ray Source	8442 reflections with *I* > 2σ(*I*)
graphite	*R*_int_ = 0.034
Detector resolution: 16.1419 pixels mm^-1^	θ_max_ = 29.6°, θ_min_ = 2.0°
ω scan	*h* = −13→13
Absorption correction: multi-scan (*CrysAlis RED*; Oxford Diffraction, 2008)	*k* = −17→17
*T*_min_ = 0.848, *T*_max_ = 1.000	*l* = −23→23
53102 measured reflections	

### Refinement

**Table:**

Refinement on *F*^2^	Primary atom site location: structure-invariant direct methods
Least-squares matrix: full	Secondary atom site location: difference Fourier map
*R*[*F*^2^ > 2σ(*F*^2^)] = 0.029	Hydrogen site location: inferred from neighbouring sites
*wR*(*F*^2^) = 0.064	H-atom parameters constrained
*S* = 0.93	*w* = 1/[σ^2^(*F*_o_^2^) + (0.0374*P*)^2^] where *P* = (*F*_o_^2^ + 2*F*_c_^2^)/3
10448 reflections	(Δ/σ)_max_ = 0.028
538 parameters	Δρ_max_ = 2.18 e Å^−^^3^
69 restraints	Δρ_min_ = −0.84 e Å^−^^3^

### Special details

**Table:**

Geometry. All e.s.d.'s (except the e.s.d. in the dihedral angle between two l.s. planes) are estimated using the full covariance matrix. The cell e.s.d.'s are taken into account individually in the estimation of e.s.d.'s in distances, angles and torsion angles; correlations between e.s.d.'s in cell parameters are only used when they are defined by crystal symmetry. An approximate (isotropic) treatment of cell e.s.d.'s is used for estimating e.s.d.'s involving l.s. planes.Least-squares planes (*x*,*y*,*z* in crystal coordinates) and deviations from them (* indicates atom used to define plane)3.2255 (0.0106) *x* + 5.5173 (0.0081) *y* + 15.8973 (0.0047) *z* = 8.4546 (0.0089)* -0.0191 (0.0010) Ir * 0.0215 (0.0015) N3 * 0.0244 (0.0015) N4 * -0.0109 (0.0018) C23 * -0.0159 (0.0018) C28Rms deviation of fitted atoms = 0.0190- 5.0032 (0.0075) *x* + 7.5842 (0.0079) *y* - 2.6742 (0.0193) *z* = 0.8875 (0.0113)Angle to previous plane (with approximate e.s.d.) = 88.07 (0.08)* -0.0017 (0.0011) Ir * -0.0003 (0.0016) N1 * 0.0030 (0.0019) C1 * -0.0050 (0.0018) C6 * 0.0041 (0.0015) C11Rms deviation of fitted atoms = 0.00338.0059 (0.0047) *x* + 7.0413 (0.0123) *y* - 6.0961 (0.0152) *z* = 9.2723 (0.0097)Angle to previous plane (with approximate e.s.d.) = 87.21 (0.07)* -0.0001 (0.0011) Ir * 0.0052 (0.0015) N2 * -0.0092 (0.0018) C12 * 0.0091 (0.0019) C17 * -0.0051 (0.0016) C22Rms deviation of fitted atoms = 0.0066- 4.9239 (0.0124) *x* + 7.5102 (0.0143) *y* - 3.5667 (0.0238) *z* = 0.6667 (0.0227)Angle to previous plane (with approximate e.s.d.) = 88.93 (0.09)* -0.0051 (0.0020) N1 * 0.0097 (0.0022) C1 * -0.0065 (0.0026) C2 * -0.0011 (0.0029) C3 * 0.0057 (0.0026) C4 * -0.0027 (0.0022) C5Rms deviation of fitted atoms = 0.0058- 5.0855 (0.0105) *x* + 7.5155 (0.0121) *y* - 2.5377 (0.0215) *z* = 0.7805 (0.0195)Angle to previous plane (with approximate e.s.d.) = 3.74 (0.23)* -0.0030 (0.0020) C6 * -0.0032 (0.0024) C7 * 0.0061 (0.0024) C8 * -0.0028 (0.0023) C9 * -0.0034 (0.0021) C10 * 0.0063 (0.0019) C11Rms deviation of fitted atoms = 0.00447.8608 (0.0075) *x* + 6.9324 (0.0128) *y* - 6.5592 (0.0188) *z* = 9.0941 (0.0078)Angle to previous plane (with approximate e.s.d.) = 87.00 (0.09)* -0.0057 (0.0019) N2 * 0.0051 (0.0020) C12 * -0.0006 (0.0023) C13 * -0.0035 (0.0023) C14 * 0.0030 (0.0022) C15 * 0.0016 (0.0021) C16Rms deviation of fitted atoms = 0.00378.1881 (0.0069) *x* + 6.6251 (0.0132) *y* - 6.0523 (0.0203) *z* = 9.0428 (0.0130)Angle to previous plane (with approximate e.s.d.) = 3.41 (0.18)* 0.0009 (0.0020) C17 * 0.0004 (0.0022) C18 * -0.0039 (0.0023) C19 * 0.0060 (0.0024) C20 * -0.0045 (0.0022) C21 * 0.0010 (0.0021) C22Rms deviation of fitted atoms = 0.00353.1752 (0.0118) *x* + 5.2258 (0.0137) *y* + 16.0284 (0.0067) *z* = 8.3218 (0.0125)Angle to previous plane (with approximate e.s.d.) = 85.49 (0.09)* -0.0036 (0.0019) N3 * -0.0039 (0.0020) C23 * 0.0076 (0.0022) C24 * -0.0040 (0.0023) C25 * -0.0033 (0.0022) C26 * 0.0073 (0.0020) C27Rms deviation of fitted atoms = 0.00533.4499 (0.0143) *x* + 5.2695 (0.0146) *y* + 15.9354 (0.0080) *z* = 8.4866 (0.0099)Angle to previous plane (with approximate e.s.d.) = 1.70 (0.14)* -0.0012 (0.0016) N4 * -0.0019 (0.0016) N5 * -0.0046 (0.0016) N6 * 0.0036 (0.0016) C28 * 0.0040 (0.0017) C29Rms deviation of fitted atoms = 0.0033
Refinement. Refinement of *F*^2^ against ALL reflections. The weighted *R*-factor *wR* and goodness of fit *S* are based on *F*^2^, conventional *R*-factors *R* are based on *F*, with *F* set to zero for negative *F*^2^. The threshold expression of *F*^2^ > σ(*F*^2^) is used only for calculating *R*-factors(gt) *etc*. and is not relevant to the choice of reflections for refinement. *R*-factors based on *F*^2^ are statistically about twice as large as those based on *F*, and *R*- factors based on ALL data will be even larger.

### Fractional atomic coordinates and isotropic or equivalent isotropic displacement parameters (Å^2^)

**Table:**

	*x*	*y*	*z*	*U*_iso_*/*U*_eq_	Occ. (<1)
Ir	0.714159 (11)	0.644989 (9)	0.161874 (7)	0.02659 (4)	
N1	0.7504 (3)	0.7041 (2)	0.26105 (15)	0.0309 (5)	
N2	0.6692 (2)	0.6055 (2)	0.05631 (14)	0.0297 (5)	
N3	0.8732 (2)	0.4767 (2)	0.19058 (14)	0.0278 (5)	
N4	0.5913 (2)	0.5443 (2)	0.22449 (14)	0.0290 (5)	
N5	0.4497 (3)	0.5607 (2)	0.24969 (15)	0.0313 (5)	
N6	0.5827 (3)	0.3722 (2)	0.28304 (14)	0.0305 (5)	
F1	1.0571 (2)	0.8804 (2)	0.19271 (14)	0.0637 (6)	
F2	1.0605 (2)	0.7954 (2)	−0.06767 (12)	0.0556 (5)	
F3	0.3418 (2)	0.9040 (2)	−0.04453 (13)	0.0608 (6)	
F4	0.3587 (2)	1.08740 (17)	0.17490 (17)	0.0701 (7)	
O	0.2211 (3)	0.1772 (2)	0.41368 (14)	0.0503 (6)	
C1	0.8454 (3)	0.7639 (3)	0.25172 (19)	0.0363 (7)	
C2	0.8715 (4)	0.8095 (3)	0.3164 (2)	0.0513 (9)	
H2	0.9397	0.8494	0.3101	0.062*	
C3	0.7979 (5)	0.7970 (4)	0.3901 (2)	0.0625 (11)	
H3	0.8147	0.8290	0.4344	0.075*	
C4	0.7006 (4)	0.7383 (3)	0.3989 (2)	0.0521 (10)	
H4	0.6489	0.7296	0.4491	0.063*	
C5	0.6797 (3)	0.6927 (3)	0.33407 (19)	0.0386 (7)	
H5	0.6130	0.6514	0.3403	0.046*	
C6	0.9075 (3)	0.7745 (2)	0.16876 (18)	0.0319 (6)	
C7	1.0059 (4)	0.8317 (3)	0.1408 (2)	0.0425 (8)	
C8	1.0578 (4)	0.8414 (3)	0.0622 (2)	0.0434 (8)	
H8	1.1240	0.8819	0.0445	0.052*	
C9	1.0087 (3)	0.7895 (3)	0.0103 (2)	0.0400 (8)	
C10	0.9111 (3)	0.7311 (3)	0.03312 (18)	0.0323 (7)	
H10	0.8807	0.6967	−0.0052	0.039*	
C11	0.8574 (3)	0.7232 (2)	0.11339 (18)	0.0280 (6)	
C12	0.5624 (3)	0.6896 (3)	0.01559 (19)	0.0351 (7)	
C13	0.5223 (4)	0.6677 (3)	−0.0547 (2)	0.0455 (9)	
H13	0.4475	0.7260	−0.0825	0.055*	
C14	0.5895 (4)	0.5627 (4)	−0.0848 (2)	0.0479 (9)	
H14	0.5614	0.5480	−0.1330	0.058*	
C15	0.6979 (4)	0.4792 (3)	−0.04409 (19)	0.0416 (8)	
H15	0.7464	0.4062	−0.0639	0.050*	
C16	0.7347 (3)	0.5033 (3)	0.02574 (18)	0.0347 (7)	
H16	0.8096	0.4455	0.0537	0.042*	
C17	0.5012 (3)	0.7966 (3)	0.05579 (19)	0.0356 (7)	
C18	0.3976 (3)	0.8979 (3)	0.0267 (2)	0.0442 (9)	
C19	0.3463 (3)	0.9952 (3)	0.0644 (3)	0.0518 (10)	
H19	0.2743	1.0633	0.0431	0.062*	
C20	0.4042 (4)	0.9899 (3)	0.1353 (3)	0.0510 (10)	
C21	0.5066 (3)	0.8936 (3)	0.1702 (2)	0.0406 (8)	
H21	0.5415	0.8944	0.2201	0.049*	
C22	0.5573 (3)	0.7946 (2)	0.12954 (19)	0.0329 (7)	
C23	0.8215 (3)	0.3924 (2)	0.22827 (16)	0.0276 (6)	
C24	0.9108 (3)	0.2797 (3)	0.24803 (19)	0.0346 (7)	
H24	0.8722	0.2226	0.2753	0.042*	
C25	1.0571 (3)	0.2515 (3)	0.2275 (2)	0.0390 (8)	
H25	1.1201	0.1742	0.2397	0.047*	
C26	1.1106 (3)	0.3365 (3)	0.18928 (19)	0.0354 (7)	
H26	1.2109	0.3185	0.1748	0.042*	
C27	1.0169 (3)	0.4480 (3)	0.17215 (17)	0.0314 (6)	
H27	1.0547	0.5065	0.1465	0.038*	
C28	0.6655 (3)	0.4329 (2)	0.24554 (16)	0.0273 (6)	
C29	0.4508 (3)	0.4553 (3)	0.28467 (17)	0.0308 (6)	
C30	0.3195 (3)	0.4309 (3)	0.32093 (18)	0.0336 (7)	
C31	0.3315 (4)	0.3168 (3)	0.35168 (18)	0.0379 (7)	
H31	0.4226	0.2569	0.3486	0.045*	
C32	0.2111 (4)	0.2905 (3)	0.38664 (19)	0.0432 (8)	
C33	0.0807 (4)	0.3776 (3)	0.3946 (2)	0.0469 (9)	
H33	−0.0007	0.3605	0.4215	0.056*	
C34	0.0688 (4)	0.4897 (3)	0.3636 (2)	0.0487 (9)	
H34	−0.0219	0.5496	0.3684	0.058*	
C35	0.1860 (3)	0.5171 (3)	0.3254 (2)	0.0429 (8)	
H35	0.1751	0.5945	0.3023	0.052*	
C36	0.3592 (7)	0.0866 (5)	0.4007 (4)	0.071 (2)	0.653 (4)
H36A	0.4299	0.0918	0.4357	0.085*	0.653 (4)
H36B	0.3973	0.0954	0.3442	0.085*	0.653 (4)
C37	0.3348 (5)	−0.0286 (3)	0.4204 (2)	0.0622 (19)	0.653 (4)
C38	0.3438 (5)	−0.0840 (3)	0.49835 (19)	0.072 (2)	0.653 (4)
H38	0.3633	−0.0482	0.5399	0.086*	0.653 (4)
C39	0.3242 (6)	−0.1918 (3)	0.51547 (16)	0.0604 (19)	0.653 (4)
H39	0.3303	−0.2296	0.5687	0.072*	0.653 (4)
C40	0.2956 (6)	−0.2442 (3)	0.4547 (2)	0.0362 (13)	0.653 (4)
C41	0.2867 (6)	−0.1888 (3)	0.37680 (19)	0.0527 (18)	0.653 (4)
H41	0.2672	−0.2246	0.3353	0.063*	0.653 (4)
C42	0.3063 (5)	−0.0810 (3)	0.35967 (17)	0.0585 (16)	0.653 (4)
H42	0.3002	−0.0432	0.3064	0.070*	0.653 (4)
C43	0.2863 (11)	−0.3593 (5)	0.4678 (4)	0.0501 (18)	0.653 (4)
H43	0.2594	−0.3860	0.4240	0.060*	0.653 (4)
C44	0.311 (2)	−0.4304 (10)	0.5326 (6)	0.080 (4)	0.653 (4)
H44A	0.3378	−0.4082	0.5782	0.096*	0.653 (4)
H44B	0.3011	−0.5051	0.5346	0.096*	0.653 (4)
C36'	0.0909 (11)	0.1407 (8)	0.4352 (7)	0.049 (3)*	0.347 (4)
H36C	0.0432	0.1676	0.4871	0.059*	0.347 (4)
H36D	0.0210	0.1747	0.3936	0.059*	0.347 (4)
C37'	0.1405 (9)	0.0112 (6)	0.4411 (5)	0.049 (3)*	0.347 (4)
C38'	0.1309 (12)	−0.0504 (9)	0.5161 (5)	0.143 (8)*	0.347 (4)
H38'	0.0859	−0.0099	0.5611	0.172*	0.347 (4)
C39'	0.1870 (14)	−0.1713 (9)	0.5251 (6)	0.103 (6)*	0.347 (4)
H39'	0.1804	−0.2134	0.5763	0.124*	0.347 (4)
C40'	0.2528 (13)	−0.2305 (6)	0.4592 (8)	0.073 (5)*	0.347 (4)
C41'	0.2625 (12)	−0.1689 (8)	0.3843 (6)	0.076 (5)*	0.347 (4)
H41'	0.3074	−0.2094	0.3393	0.091*	0.347 (4)
C42'	0.2063 (10)	−0.0480 (8)	0.3753 (4)	0.078 (4)*	0.347 (4)
H42'	0.2129	−0.0059	0.3240	0.093*	0.347 (4)
C43'	0.325 (3)	−0.3545 (13)	0.4677 (15)	0.087 (7)*	0.347 (4)
H43'	0.3818	−0.3868	0.4220	0.105*	0.347 (4)
C44'	0.321 (5)	−0.427 (3)	0.5311 (19)	0.115 (11)*	0.347 (4)
H44C	0.2657	−0.3991	0.5785	0.137*	0.347 (4)
H44D	0.3729	−0.5074	0.5302	0.137*	0.347 (4)
O99	0.6874 (3)	0.1238 (2)	0.30300 (14)	0.0464 (6)	
H99	0.6500	0.1956	0.3065	0.070*	
C99	0.6872 (4)	0.1023 (3)	0.2235 (2)	0.0543 (10)	
H99A	0.5892	0.1373	0.2057	0.081*	
H99B	0.7200	0.0181	0.2207	0.081*	
H99C	0.7521	0.1363	0.1887	0.081*	

### Atomic displacement parameters (Å^2^)

**Table:**

	*U*^11^	*U*^22^	*U*^33^	*U*^12^	*U*^13^	*U*^23^
Ir	0.02141 (6)	0.02302 (6)	0.02931 (6)	−0.00108 (4)	0.00057 (4)	−0.00128 (4)
N1	0.0286 (13)	0.0270 (12)	0.0314 (13)	−0.0031 (10)	0.0062 (10)	−0.0068 (10)
N2	0.0231 (12)	0.0330 (13)	0.0301 (13)	−0.0088 (10)	−0.0002 (10)	0.0029 (10)
N3	0.0265 (12)	0.0274 (12)	0.0243 (12)	−0.0014 (10)	−0.0016 (10)	−0.0058 (10)
N4	0.0239 (12)	0.0281 (12)	0.0303 (13)	−0.0033 (10)	0.0020 (10)	−0.0047 (10)
N5	0.0249 (12)	0.0309 (13)	0.0327 (13)	−0.0040 (10)	0.0049 (10)	−0.0045 (11)
N6	0.0328 (13)	0.0285 (13)	0.0276 (13)	−0.0063 (11)	0.0008 (10)	−0.0063 (10)
F1	0.0689 (15)	0.0842 (17)	0.0595 (14)	−0.0496 (13)	0.0101 (11)	−0.0284 (12)
F2	0.0598 (13)	0.0782 (15)	0.0380 (11)	−0.0407 (12)	0.0111 (9)	−0.0034 (10)
F3	0.0347 (11)	0.0685 (15)	0.0649 (14)	−0.0097 (10)	−0.0156 (10)	0.0293 (12)
F4	0.0377 (12)	0.0281 (10)	0.130 (2)	0.0050 (9)	0.0069 (12)	−0.0134 (12)
O	0.0573 (16)	0.0502 (15)	0.0440 (14)	−0.0271 (13)	0.0128 (12)	0.0028 (12)
C1	0.0388 (17)	0.0300 (16)	0.0372 (17)	−0.0073 (14)	0.0014 (14)	−0.0077 (13)
C2	0.069 (3)	0.048 (2)	0.044 (2)	−0.0274 (19)	0.0065 (18)	−0.0164 (17)
C3	0.096 (3)	0.057 (2)	0.043 (2)	−0.033 (2)	0.010 (2)	−0.0255 (19)
C4	0.071 (3)	0.042 (2)	0.0359 (19)	−0.0122 (19)	0.0165 (18)	−0.0122 (16)
C5	0.0410 (18)	0.0311 (16)	0.0369 (18)	−0.0055 (14)	0.0063 (14)	−0.0054 (14)
C6	0.0295 (15)	0.0282 (15)	0.0336 (16)	−0.0035 (12)	−0.0008 (12)	−0.0059 (12)
C7	0.0404 (18)	0.0448 (19)	0.047 (2)	−0.0172 (15)	0.0001 (15)	−0.0161 (16)
C8	0.0368 (18)	0.048 (2)	0.050 (2)	−0.0239 (16)	0.0080 (15)	−0.0068 (16)
C9	0.0335 (17)	0.0463 (19)	0.0365 (18)	−0.0123 (15)	0.0043 (14)	−0.0009 (15)
C10	0.0277 (15)	0.0326 (16)	0.0318 (16)	−0.0048 (13)	−0.0027 (12)	−0.0010 (13)
C11	0.0180 (13)	0.0250 (14)	0.0345 (16)	0.0005 (11)	−0.0019 (11)	−0.0009 (12)
C12	0.0235 (14)	0.0431 (18)	0.0361 (17)	−0.0141 (13)	0.0002 (12)	0.0097 (14)
C13	0.0363 (18)	0.066 (2)	0.0328 (18)	−0.0220 (17)	−0.0084 (14)	0.0149 (17)
C14	0.046 (2)	0.078 (3)	0.0270 (17)	−0.032 (2)	0.0020 (15)	−0.0029 (17)
C15	0.0393 (18)	0.057 (2)	0.0326 (17)	−0.0235 (17)	0.0059 (14)	−0.0073 (15)
C16	0.0341 (16)	0.0377 (17)	0.0314 (16)	−0.0122 (14)	0.0007 (13)	−0.0031 (13)
C17	0.0204 (14)	0.0355 (16)	0.0441 (18)	−0.0086 (12)	−0.0003 (13)	0.0146 (14)
C18	0.0219 (15)	0.046 (2)	0.056 (2)	−0.0112 (14)	−0.0027 (14)	0.0210 (17)
C19	0.0171 (15)	0.0383 (19)	0.085 (3)	−0.0022 (14)	−0.0017 (17)	0.0209 (19)
C20	0.0277 (17)	0.0284 (17)	0.088 (3)	−0.0052 (14)	0.0141 (18)	−0.0027 (18)
C21	0.0200 (14)	0.0293 (16)	0.065 (2)	−0.0011 (12)	0.0024 (14)	−0.0042 (15)
C22	0.0217 (14)	0.0242 (14)	0.0462 (18)	−0.0054 (12)	0.0058 (13)	0.0048 (13)
C23	0.0273 (14)	0.0277 (14)	0.0245 (14)	−0.0049 (12)	0.0012 (11)	−0.0055 (11)
C24	0.0350 (17)	0.0254 (15)	0.0373 (17)	−0.0033 (13)	0.0004 (13)	−0.0032 (13)
C25	0.0361 (17)	0.0276 (15)	0.0423 (19)	0.0033 (13)	−0.0005 (14)	−0.0056 (14)
C26	0.0261 (15)	0.0337 (16)	0.0366 (17)	0.0019 (13)	0.0016 (13)	−0.0057 (13)
C27	0.0278 (15)	0.0331 (16)	0.0302 (16)	−0.0060 (13)	0.0006 (12)	−0.0056 (12)
C28	0.0309 (15)	0.0255 (14)	0.0230 (14)	−0.0057 (12)	0.0013 (11)	−0.0063 (11)
C29	0.0295 (15)	0.0316 (15)	0.0279 (15)	−0.0062 (13)	0.0018 (12)	−0.0056 (12)
C30	0.0331 (16)	0.0379 (17)	0.0293 (16)	−0.0115 (14)	0.0016 (13)	−0.0059 (13)
C31	0.0436 (18)	0.0378 (17)	0.0288 (16)	−0.0116 (14)	0.0020 (13)	−0.0011 (13)
C32	0.056 (2)	0.046 (2)	0.0288 (17)	−0.0224 (18)	0.0034 (15)	−0.0023 (15)
C33	0.050 (2)	0.058 (2)	0.0375 (19)	−0.0283 (19)	0.0085 (16)	−0.0067 (17)
C34	0.0362 (19)	0.049 (2)	0.056 (2)	−0.0108 (16)	0.0059 (16)	−0.0080 (18)
C35	0.0360 (18)	0.0375 (18)	0.052 (2)	−0.0119 (15)	0.0064 (15)	−0.0042 (16)
C36	0.102 (5)	0.037 (3)	0.062 (4)	−0.024 (3)	0.041 (4)	−0.008 (3)
C37	0.099 (5)	0.034 (3)	0.046 (3)	−0.021 (3)	0.027 (3)	−0.008 (2)
C38	0.141 (7)	0.052 (4)	0.034 (3)	−0.052 (4)	0.011 (4)	−0.011 (3)
C39	0.122 (6)	0.042 (3)	0.022 (3)	−0.035 (3)	−0.007 (3)	0.002 (2)
C40	0.053 (4)	0.033 (3)	0.025 (3)	−0.017 (2)	0.000 (2)	−0.0018 (18)
C41	0.072 (4)	0.045 (3)	0.029 (3)	−0.005 (3)	0.000 (3)	−0.004 (2)
C42	0.070 (4)	0.040 (3)	0.046 (3)	0.003 (3)	0.005 (3)	−0.001 (2)
C43	0.077 (6)	0.050 (3)	0.035 (3)	−0.031 (3)	−0.011 (3)	−0.009 (2)
C44	0.170 (11)	0.059 (5)	0.039 (4)	−0.079 (6)	−0.002 (4)	0.002 (3)
O99	0.0594 (16)	0.0366 (13)	0.0406 (13)	−0.0142 (12)	−0.0046 (11)	0.0005 (10)
C99	0.073 (3)	0.044 (2)	0.050 (2)	−0.0206 (19)	−0.0161 (19)	−0.0038 (17)

### Geometric parameters (Å, °)

**Table:**

Ir—C11	2.010 (3)	C24—C25	1.381 (4)
Ir—C22	2.012 (3)	C24—H24	0.9500
Ir—N1	2.033 (2)	C25—C26	1.377 (5)
Ir—N2	2.049 (2)	C25—H25	0.9500
Ir—N4	2.118 (2)	C26—C27	1.379 (4)
Ir—N3	2.158 (2)	C26—H26	0.9500
N1—C5	1.359 (4)	C27—H27	0.9500
N1—C1	1.361 (4)	C29—C30	1.476 (4)
N2—C16	1.348 (4)	C30—C35	1.384 (4)
N2—C12	1.370 (4)	C30—C31	1.395 (4)
N3—C27	1.352 (4)	C31—C32	1.385 (5)
N3—C23	1.356 (4)	C31—H31	0.9500
N4—C28	1.332 (3)	C32—C33	1.373 (5)
N4—N5	1.370 (3)	C33—C34	1.373 (5)
N5—C29	1.342 (4)	C33—H33	0.9500
N6—C28	1.339 (4)	C34—C35	1.380 (5)
N6—C29	1.355 (4)	C34—H34	0.9500
F1—C7	1.359 (4)	C35—H35	0.9500
F2—C9	1.357 (4)	C36—C37	1.503 (6)
F3—C18	1.363 (4)	C36—H36A	0.9900
F4—C20	1.371 (4)	C36—H36B	0.9900
O—C32	1.375 (4)	C37—C38	1.3900
O—C36	1.454 (6)	C37—C42	1.3900
O—C36'	1.495 (11)	C38—C39	1.3900
C1—C2	1.385 (5)	C38—H38	0.9500
C1—C6	1.469 (4)	C39—C40	1.3900
C2—C3	1.383 (5)	C39—H39	0.9500
C2—H2	0.9500	C40—C41	1.3900
C3—C4	1.372 (6)	C40—C43	1.435 (6)
C3—H3	0.9500	C41—C42	1.3900
C4—C5	1.367 (5)	C41—H41	0.9500
C4—H4	0.9500	C42—H42	0.9500
C5—H5	0.9500	C43—C44	1.290 (9)
C6—C7	1.390 (4)	C43—H43	0.9500
C6—C11	1.418 (4)	C44—H44A	0.9500
C7—C8	1.371 (5)	C44—H44B	0.9500
C8—C9	1.373 (5)	C36'—C37'	1.490 (10)
C8—H8	0.9500	C36'—H36C	0.9900
C9—C10	1.381 (4)	C36'—H36D	0.9900
C10—C11	1.400 (4)	C37'—C38'	1.3900
C10—H10	0.9500	C37'—C42'	1.3900
C12—C13	1.383 (5)	C38'—C39'	1.3900
C12—C17	1.475 (5)	C38'—H38'	0.9500
C13—C14	1.376 (5)	C39'—C40'	1.3900
C13—H13	0.9500	C39'—H39'	0.9500
C14—C15	1.376 (5)	C40'—C41'	1.3900
C14—H14	0.9500	C40'—C43'	1.439 (14)
C15—C16	1.374 (4)	C41'—C42'	1.3900
C15—H15	0.9500	C41'—H41'	0.9500
C16—H16	0.9500	C42'—H42'	0.9500
C17—C18	1.382 (4)	C43'—C44'	1.296 (16)
C17—C22	1.416 (5)	C43'—H43'	0.9500
C18—C19	1.351 (5)	C44'—H44C	0.9500
C19—C20	1.370 (6)	C44'—H44D	0.9500
C19—H19	0.9500	O99—C99	1.406 (4)
C20—C21	1.377 (5)	O99—H99	0.8400
C21—C22	1.395 (4)	C99—H99A	0.9800
C21—H21	0.9500	C99—H99B	0.9800
C23—C24	1.382 (4)	C99—H99C	0.9800
C23—C28	1.457 (4)		
			
C11—Ir—C22	87.62 (11)	C25—C24—H24	120.5
C11—Ir—N1	80.20 (11)	C23—C24—H24	120.5
C22—Ir—N1	93.29 (12)	C26—C25—C24	119.3 (3)
C11—Ir—N2	96.44 (11)	C26—C25—H25	120.3
C22—Ir—N2	80.71 (12)	C24—C25—H25	120.3
N1—Ir—N2	173.28 (9)	C25—C26—C27	119.3 (3)
C11—Ir—N4	170.49 (10)	C25—C26—H26	120.3
C22—Ir—N4	101.08 (10)	C27—C26—H26	120.3
N1—Ir—N4	95.35 (10)	N3—C27—C26	122.2 (3)
N2—Ir—N4	88.81 (9)	N3—C27—H27	118.9
C11—Ir—N3	95.37 (10)	C26—C27—H27	118.9
C22—Ir—N3	174.78 (11)	N4—C28—N6	113.2 (2)
N1—Ir—N3	91.44 (9)	N4—C28—C23	118.7 (3)
N2—Ir—N3	94.68 (9)	N6—C28—C23	128.1 (3)
N4—Ir—N3	76.25 (9)	N5—C29—N6	114.3 (3)
C5—N1—C1	118.9 (3)	N5—C29—C30	123.2 (3)
C5—N1—Ir	123.7 (2)	N6—C29—C30	122.5 (3)
C1—N1—Ir	117.3 (2)	C35—C30—C31	119.2 (3)
C16—N2—C12	118.7 (3)	C35—C30—C29	122.3 (3)
C16—N2—Ir	124.8 (2)	C31—C30—C29	118.5 (3)
C12—N2—Ir	116.5 (2)	C32—C31—C30	120.2 (3)
C27—N3—C23	118.1 (2)	C32—C31—H31	119.9
C27—N3—Ir	125.9 (2)	C30—C31—H31	119.9
C23—N3—Ir	115.98 (18)	C33—C32—O	119.6 (3)
C28—N4—N5	107.2 (2)	C33—C32—C31	120.0 (3)
C28—N4—Ir	115.38 (18)	O—C32—C31	120.4 (3)
N5—N4—Ir	137.37 (18)	C32—C33—C34	119.6 (3)
C29—N5—N4	103.8 (2)	C32—C33—H33	120.2
C28—N6—C29	101.5 (2)	C34—C33—H33	120.2
C32—O—C36	118.1 (3)	C33—C34—C35	121.3 (3)
C32—O—C36'	122.2 (4)	C33—C34—H34	119.3
C36—O—C36'	117.7 (5)	C35—C34—H34	119.3
N1—C1—C2	120.2 (3)	C30—C35—C34	119.5 (3)
N1—C1—C6	112.8 (3)	C30—C35—H35	120.3
C2—C1—C6	127.0 (3)	C34—C35—H35	120.3
C3—C2—C1	119.8 (4)	O—C36—C37	107.5 (4)
C3—C2—H2	120.1	O—C36—H36A	110.2
C1—C2—H2	120.1	C37—C36—H36A	110.2
C4—C3—C2	119.8 (4)	O—C36—H36B	110.2
C4—C3—H3	120.1	C37—C36—H36B	110.2
C2—C3—H3	120.1	H36A—C36—H36B	108.5
C5—C4—C3	118.6 (3)	C38—C37—C42	120.0
C5—C4—H4	120.7	C38—C37—C36	120.6 (4)
C3—C4—H4	120.7	C42—C37—C36	119.4 (4)
N1—C5—C4	122.6 (3)	C37—C38—C39	120.0
N1—C5—H5	118.7	C37—C38—H38	120.0
C4—C5—H5	118.7	C39—C38—H38	120.0
C7—C6—C11	118.7 (3)	C40—C39—C38	120.0
C7—C6—C1	126.0 (3)	C40—C39—H39	120.0
C11—C6—C1	115.4 (3)	C38—C39—H39	120.0
F1—C7—C8	116.5 (3)	C39—C40—C41	120.0
F1—C7—C6	120.0 (3)	C39—C40—C43	122.2 (3)
C8—C7—C6	123.5 (3)	C41—C40—C43	117.6 (3)
C7—C8—C9	116.4 (3)	C42—C41—C40	120.0
C7—C8—H8	121.8	C42—C41—H41	120.0
C9—C8—H8	121.8	C40—C41—H41	120.0
F2—C9—C8	117.8 (3)	C41—C42—C37	120.0
F2—C9—C10	118.5 (3)	C41—C42—H42	120.0
C8—C9—C10	123.7 (3)	C37—C42—H42	120.0
C9—C10—C11	119.4 (3)	C44—C43—C40	127.1 (7)
C9—C10—H10	120.3	C44—C43—H43	116.5
C11—C10—H10	120.3	C40—C43—H43	116.5
C10—C11—C6	118.3 (3)	C43—C44—H44A	120.0
C10—C11—Ir	127.3 (2)	C43—C44—H44B	120.0
C6—C11—Ir	114.3 (2)	H44A—C44—H44B	120.0
N2—C12—C13	119.8 (3)	C37'—C36'—O	107.3 (7)
N2—C12—C17	112.7 (3)	C37'—C36'—H36C	110.2
C13—C12—C17	127.5 (3)	O—C36'—H36C	110.2
C14—C13—C12	120.8 (3)	C37'—C36'—H36D	110.2
C14—C13—H13	119.6	O—C36'—H36D	110.2
C12—C13—H13	119.6	H36C—C36'—H36D	108.5
C15—C14—C13	119.0 (3)	C38'—C37'—C42'	120.0
C15—C14—H14	120.5	C38'—C37'—C36'	118.3 (7)
C13—C14—H14	120.5	C42'—C37'—C36'	121.4 (7)
C16—C15—C14	118.8 (3)	C37'—C38'—C39'	120.0
C16—C15—H15	120.6	C37'—C38'—H38'	120.0
C14—C15—H15	120.6	C39'—C38'—H38'	120.0
N2—C16—C15	122.9 (3)	C40'—C39'—C38'	120.0
N2—C16—H16	118.6	C40'—C39'—H39'	120.0
C15—C16—H16	118.6	C38'—C39'—H39'	120.0
C18—C17—C22	118.1 (3)	C39'—C40'—C41'	120.0
C18—C17—C12	125.6 (3)	C39'—C40'—C43'	121.5 (11)
C22—C17—C12	116.2 (3)	C41'—C40'—C43'	118.2 (11)
C19—C18—F3	115.9 (3)	C42'—C41'—C40'	120.0
C19—C18—C17	124.0 (4)	C42'—C41'—H41'	120.0
F3—C18—C17	120.1 (3)	C40'—C41'—H41'	120.0
C18—C19—C20	116.0 (3)	C41'—C42'—C37'	120.0
C18—C19—H19	122.0	C41'—C42'—H42'	120.0
C20—C19—H19	122.0	C37'—C42'—H42'	120.0
C19—C20—F4	118.3 (3)	C44'—C43'—C40'	127 (3)
C19—C20—C21	125.0 (4)	C44'—C43'—H43'	116.5
F4—C20—C21	116.7 (4)	C40'—C43'—H43'	116.6
C20—C21—C22	117.5 (4)	C43'—C44'—H44C	119.9
C20—C21—H21	121.2	C43'—C44'—H44D	120.1
C22—C21—H21	121.2	H44C—C44'—H44D	120.0
C21—C22—C17	119.4 (3)	C99—O99—H99	109.5
C21—C22—Ir	126.6 (3)	O99—C99—H99A	109.5
C17—C22—Ir	113.9 (2)	O99—C99—H99B	109.5
N3—C23—C24	122.1 (3)	H99A—C99—H99B	109.5
N3—C23—C28	113.5 (2)	O99—C99—H99C	109.5
C24—C23—C28	124.4 (3)	H99A—C99—H99C	109.5
C25—C24—C23	119.0 (3)	H99B—C99—H99C	109.5
			
C11—Ir—N1—C5	176.2 (2)	C18—C19—C20—F4	178.2 (3)
C22—Ir—N1—C5	89.2 (2)	C18—C19—C20—C21	−1.2 (5)
N4—Ir—N1—C5	−12.3 (2)	C19—C20—C21—C22	1.3 (5)
N3—Ir—N1—C5	−88.6 (2)	F4—C20—C21—C22	−178.2 (3)
C11—Ir—N1—C1	0.0 (2)	C20—C21—C22—C17	−0.7 (4)
C22—Ir—N1—C1	−87.0 (2)	C20—C21—C22—Ir	176.1 (2)
N4—Ir—N1—C1	171.5 (2)	C18—C17—C22—C21	0.2 (4)
N3—Ir—N1—C1	95.2 (2)	C12—C17—C22—C21	178.7 (3)
C11—Ir—N2—C16	94.2 (2)	C18—C17—C22—Ir	−177.0 (2)
C22—Ir—N2—C16	−179.3 (2)	C12—C17—C22—Ir	1.5 (3)
N4—Ir—N2—C16	−77.9 (2)	C11—Ir—C22—C21	−80.6 (3)
N3—Ir—N2—C16	−1.8 (2)	N1—Ir—C22—C21	−0.6 (3)
C11—Ir—N2—C12	−87.1 (2)	N2—Ir—C22—C21	−177.5 (3)
C22—Ir—N2—C12	−0.6 (2)	N4—Ir—C22—C21	95.6 (3)
N4—Ir—N2—C12	100.8 (2)	C11—Ir—C22—C17	96.4 (2)
N3—Ir—N2—C12	176.9 (2)	N1—Ir—C22—C17	176.4 (2)
C11—Ir—N3—C27	−3.8 (2)	N2—Ir—C22—C17	−0.5 (2)
N1—Ir—N3—C27	−84.0 (2)	N4—Ir—C22—C17	−87.5 (2)
N2—Ir—N3—C27	93.2 (2)	C27—N3—C23—C24	0.1 (4)
N4—Ir—N3—C27	−179.2 (2)	Ir—N3—C23—C24	178.0 (2)
C11—Ir—N3—C23	178.4 (2)	C27—N3—C23—C28	179.8 (2)
N1—Ir—N3—C23	98.2 (2)	Ir—N3—C23—C28	−2.3 (3)
N2—Ir—N3—C23	−84.6 (2)	N3—C23—C24—C25	−1.1 (5)
N4—Ir—N3—C23	3.00 (19)	C28—C23—C24—C25	179.2 (3)
C22—Ir—N4—C28	172.1 (2)	C23—C24—C25—C26	1.1 (5)
N1—Ir—N4—C28	−93.5 (2)	C24—C25—C26—C27	−0.1 (5)
N2—Ir—N4—C28	91.8 (2)	C23—N3—C27—C26	1.0 (4)
N3—Ir—N4—C28	−3.30 (19)	Ir—N3—C27—C26	−176.7 (2)
C22—Ir—N4—N5	−6.3 (3)	C25—C26—C27—N3	−1.0 (5)
N1—Ir—N4—N5	88.2 (3)	N5—N4—C28—N6	0.5 (3)
N2—Ir—N4—N5	−86.5 (3)	Ir—N4—C28—N6	−178.33 (18)
N3—Ir—N4—N5	178.3 (3)	N5—N4—C28—C23	−177.9 (2)
C28—N4—N5—C29	0.1 (3)	Ir—N4—C28—C23	3.3 (3)
Ir—N4—N5—C29	178.5 (2)	C29—N6—C28—N4	−0.8 (3)
C5—N1—C1—C2	1.6 (4)	C29—N6—C28—C23	177.4 (3)
Ir—N1—C1—C2	178.0 (2)	N3—C23—C28—N4	−0.7 (4)
C5—N1—C1—C6	−176.8 (2)	C24—C23—C28—N4	179.0 (3)
Ir—N1—C1—C6	−0.4 (3)	N3—C23—C28—N6	−178.8 (3)
N1—C1—C2—C3	−1.7 (5)	C24—C23—C28—N6	0.9 (5)
C6—C1—C2—C3	176.4 (3)	N4—N5—C29—N6	−0.6 (3)
C1—C2—C3—C4	0.7 (6)	N4—N5—C29—C30	−179.8 (3)
C2—C3—C4—C5	0.5 (6)	C28—N6—C29—N5	0.9 (3)
C1—N1—C5—C4	−0.4 (5)	C28—N6—C29—C30	−180.0 (3)
Ir—N1—C5—C4	−176.6 (2)	N5—C29—C30—C35	−3.6 (5)
C3—C4—C5—N1	−0.6 (5)	N6—C29—C30—C35	177.3 (3)
N1—C1—C6—C7	179.3 (3)	N5—C29—C30—C31	176.4 (3)
C2—C1—C6—C7	1.0 (5)	N6—C29—C30—C31	−2.7 (4)
N1—C1—C6—C11	0.8 (4)	C35—C30—C31—C32	−0.4 (5)
C2—C1—C6—C11	−177.5 (3)	C29—C30—C31—C32	179.6 (3)
C11—C6—C7—F1	−179.2 (3)	C36—O—C32—C33	177.1 (4)
C1—C6—C7—F1	2.5 (5)	C36'—O—C32—C33	13.7 (7)
C11—C6—C7—C8	0.1 (5)	C36—O—C32—C31	−3.1 (6)
C1—C6—C7—C8	−178.3 (3)	C36'—O—C32—C31	−166.5 (5)
F1—C7—C8—C9	178.4 (3)	C30—C31—C32—C33	−3.2 (5)
C6—C7—C8—C9	−0.9 (5)	C30—C31—C32—O	177.0 (3)
C7—C8—C9—F2	−178.5 (3)	O—C32—C33—C34	−176.3 (3)
C7—C8—C9—C10	0.8 (5)	C31—C32—C33—C34	3.9 (5)
F2—C9—C10—C11	179.4 (3)	C32—C33—C34—C35	−1.0 (6)
C8—C9—C10—C11	0.0 (5)	C31—C30—C35—C34	3.2 (5)
C9—C10—C11—C6	−0.9 (4)	C29—C30—C35—C34	−176.8 (3)
C9—C10—C11—Ir	179.4 (2)	C33—C34—C35—C30	−2.5 (6)
C7—C6—C11—C10	0.8 (4)	C32—O—C36—C37	−169.1 (3)
C1—C6—C11—C10	179.4 (3)	C36'—O—C36—C37	−5.0 (8)
C7—C6—C11—Ir	−179.4 (2)	O—C36—C37—C38	−88.2 (5)
C1—C6—C11—Ir	−0.9 (3)	O—C36—C37—C42	93.2 (5)
C22—Ir—C11—C10	−86.0 (3)	C42—C37—C38—C39	0.0
N1—Ir—C11—C10	−179.8 (3)	C36—C37—C38—C39	−178.6 (4)
N2—Ir—C11—C10	−5.7 (3)	C37—C38—C39—C40	0.0
N3—Ir—C11—C10	89.7 (2)	C38—C39—C40—C41	0.0
C22—Ir—C11—C6	94.2 (2)	C38—C39—C40—C43	174.8 (7)
N1—Ir—C11—C6	0.49 (19)	C39—C40—C41—C42	0.0
N2—Ir—C11—C6	174.6 (2)	C43—C40—C41—C42	−175.0 (6)
N3—Ir—C11—C6	−90.0 (2)	C40—C41—C42—C37	0.0
C16—N2—C12—C13	1.1 (4)	C38—C37—C42—C41	0.0
Ir—N2—C12—C13	−177.7 (2)	C36—C37—C42—C41	178.6 (4)
C16—N2—C12—C17	−179.7 (2)	C39—C40—C43—C44	−4.5 (17)
Ir—N2—C12—C17	1.5 (3)	C41—C40—C43—C44	170.4 (13)
N2—C12—C13—C14	−0.7 (5)	C32—O—C36'—C37'	165.6 (5)
C17—C12—C13—C14	−179.7 (3)	C36—O—C36'—C37'	2.1 (9)
C12—C13—C14—C15	−0.2 (5)	O—C36'—C37'—C38'	112.4 (7)
C13—C14—C15—C16	0.5 (5)	O—C36'—C37'—C42'	−62.2 (10)
C12—N2—C16—C15	−0.8 (4)	C42'—C37'—C38'—C39'	0.0
Ir—N2—C16—C15	177.9 (2)	C36'—C37'—C38'—C39'	−174.7 (9)
C14—C15—C16—N2	0.0 (5)	C37'—C38'—C39'—C40'	0.0
N2—C12—C17—C18	176.4 (3)	C38'—C39'—C40'—C41'	0.0
C13—C12—C17—C18	−4.5 (5)	C38'—C39'—C40'—C43'	173.5 (15)
N2—C12—C17—C22	−1.9 (4)	C39'—C40'—C41'—C42'	0.0
C13—C12—C17—C22	177.1 (3)	C43'—C40'—C41'—C42'	−173.8 (14)
C22—C17—C18—C19	−0.2 (5)	C40'—C41'—C42'—C37'	0.0
C12—C17—C18—C19	−178.5 (3)	C38'—C37'—C42'—C41'	0.0
C22—C17—C18—F3	179.3 (3)	C36'—C37'—C42'—C41'	174.5 (9)
C12—C17—C18—F3	1.0 (5)	C39'—C40'—C43'—C44'	11 (4)
F3—C18—C19—C20	−178.9 (3)	C41'—C40'—C43'—C44'	−175 (3)
C17—C18—C19—C20	0.7 (5)		

### Hydrogen-bond geometry (Å, °)

**Table:**

*D*—H···*A*	*D*—H	H···*A*	*D*···*A*	*D*—H···*A*
O99—H99···N6	0.84	2.04	2.853 (3)	164
C24—H24···O99	0.95	2.49	3.368 (4)	154
C31—H31···O99	0.95	2.66	3.587 (4)	164
C8—H8···F3^i^	0.95	2.55	3.412 (4)	151
C25—H25···F4^ii^	0.95	2.43	3.088 (4)	126
C3—H3···O^iii^	0.95	2.54	3.353 (5)	143
C14—H14···N5^iv^	0.95	2.57	3.475 (5)	160
C39—H39···O99^v^	0.95	2.64	3.272 (4)	124

Symmetry codes: (i) *x*+1, *y*, *z*; (ii) *x*+1, *y*−1, *z*; (iii) −*x*+1, −*y*+1, −*z*+1; (iv) −*x*+1, −*y*+1, −*z*; (v) −*x*+1, −*y*, −*z*+1.
